# Clinical trial to evaluate the performance of a flexible self-adherent absorbent dressing coated with a soft silicone layer compared to a standard wound dressing after orthopedic or spinal surgery: study protocol for a randomized controlled trial

**DOI:** 10.1186/s13063-015-0599-z

**Published:** 2015-03-07

**Authors:** Jan Bredow, Johannes Oppermann, Katharina Hoffmann, Martin Hellmich, Birte Wenk, Marvin Simons, Peer Eysel, Kourosh Zarghooni

**Affiliations:** Department of Orthopedics and Trauma Surgery, University Hospital of Cologne, Kerpener Str. 62, Cologne, D - 50924 Germany; Clinical Trials Centre, BMBF 01KN0706, University of Cologne, Kerpener Str. 62, Cologne, 50924 Germany; Institute of Medical Statistics, Informatics and Epidemiology, University of Cologne, Kerpener Str. 62, Cologne, 50924 Germany

**Keywords:** wound blistering, wound infection, dressing

## Abstract

**Background:**

Postoperative wound infection is a preventable risk. One potential postoperative complication is blistering, which leads to increased pain, delayed healing, and higher care costs. The incidence of wound blisters has been reported to be between 6 and 24%. The aim of this study is to assess whether the risks of postoperative blistering and wound infections within the first 6 days postsurgery will be reduced using a special dressing compared to a standard one.

**Methods/Design:**

This is a randomized clinical trial in a University hospital. Patients presenting for knee or hip arthroplasty or spine procedures will be assessed against study inclusion and exclusion criteria. After giving written informed consent, patients will be randomized to participate in the 7-day study during hospitalization. One hundred patients will be randomized per group. The primary outcome measure is blistering incidence from day 0 to day 6 postsurgery. Photo documentation will be evaluated in a blinded manner by the Clinical Evaluation Committee (CEC).

**Discussion:**

A new dressing will be compared to the standard wound dressing regarding the risk of postoperative blistering, wound infection, and patient comfort. This study will assess the potential advantages of a modern wound dressing.

**Trial registration:**

ClinicalTrials.gov identifier NCT01988818 (Entered 13 November 2011).

## Background

In view of an increasing elderly population, several authors expect further increases of joint replacement and spine procedures in western industrialized nations [1-3]. One postoperative complication is wound blistering, which leads to increased pain, delayed healing, and increased susceptibility to wound infection due to compromised skin integrity. Blistering occurs when the dermis is separated from the epidermis, and is the invariable result of continuous abrasion. The deep, finger-like projections of epidermal tissue holding the epidermis and dermis together are weakened, allowing the two skin layers to separate. Most blistering occurs on the fifth or sixth postoperative day [4].

There are several other factors that influence the development of blistering. These include the skin changes generally evident in older patients: that is, there is less production of collagen (a tough, fibrous protein that makes skin strong) and elastin (which makes skin flexible). Collagen production decreases significantly with age after the fifth decade [5].

The Incidence of wound blistering has been reported in the literature to be between 6 and 24% [4,6,7]. Postoperative wound complications and surgical site infections can increase recovery times, inpatient care costs, and morbidity rates [8].

Mepilex^®^ Border Post-Op is a highly conformable self-adherent dressing that absorbs blood exudates and should minimize the risk of maceration. The adhesive uses Safetac^TM^ technology, a unique and patented soft silicone adhesive technology that is designed to minimize pain as well as trauma to the wound and surrounding skin. Mepilex Border Post-Op is CE marked on the equivalent device Mepilex^®^ Boarder.

In a recent randomized clinical study, three types of dressings (Mepore Pro, Mepilex Border, and Hypafix Transparent) were compared in 150 consecutive hip surgery subjects regarding the occurrence of tape blisters. Blister prevalence was significantly lower for the Mepilex Border group (3%) than for the Mepore Pro (59%, *P* <0.01) and Hypafix group (61%, *P* <0.01). The mean time between surgery and blister occurrence and the total number of dressings used during hospital stay were also significantly lower for the Mepilex Border group compared to the Mepore Pro and Hypafix groups (*P* <0.01). In summary, the dressing with a silicone adhesive (Mepilex Border) significantly reduced the prevalence of blisters following hip surgery [9].

The overall rationale for the current trial is to evaluate the clinical performance of Mepilex Border Post-Op regarding the risks of blistering and maceration as well as the need for dressing changes, due to its high absorptive capacity compared to standard wound dressings. In addition, its performance after spine surgery will be evaluated. To our knowledge, it is the first randomized clinical trial with modern wound dressings for spine surgery at this stage of development.

### Objective

The primary objective of this clinical trial is to evaluate the performance of a self-adhesive absorbent postoperative dressing coated with a soft silicone layer in minimizing the risk of blistering compared to the hospital standard dressing (Cosmopor E^TM^, Fa. Hartmann) after hip and knee arthroplasty or spinal surgery. The study will be conducted in a clinical care setting at a University Hospital. Secondary objectives are to evaluate dressing performance, comfort, conformability and overall acceptability, pain before, during, and after dressing removal, overall cost regarding dressing wear time, time for dressing changes, and required personnel resources. Wound blisters will be documented by photographing and counting the number of blisters and its size. The documentation is done by one member of our Clinical Evaluation Committee (CEC) during the whole period of the study. The evaluation will be done separately by different members of our CEC in a blinded manner.

## Methods/Design

The study is designed as a randomized trial in a clinical care setting at a University hospital with two parallel groups. Patients presenting for knee or hip arthroplasty or spine surgery will be assessed according to study inclusion and exclusion criteria. Hip and knee arthroplasty are often done in standardized operation procedures. In spine surgery, there is a large variety of operations. We decide to standardize this by excluding operations with a hospital stay of fewer than 6 days, infections, tumor operations or revisions in general. After written informed consent, the patients will be randomized to participate in the 7-day study. The research in this trial will be performed with the approval of the Ethics Committee of the Medical Faculty of the University Hospital of Cologne under the reference number 13-348. The study is performed monocentrally, so there is no other Ethics Committee involved. Research carried out in the trial will be in compliance with the Helsinki Declaration.

### Participants and recruitment

Patients aged 18 years or older presenting for hip or knee arthroplasty or spine surgery and who will stay longer than 6 days in-house, are eligible for the trial. Patients whose wounds cannot be appropriately covered with the dressing will be excluded from this study.

Inclusion criteria are as follow:

1. Age ≥18 years
2. An expected total length of inpatient stay of 6 or more days
3. Undergoing elective primary arthroplasty of the hip or knee or spinal surgery
4. Undergoing hip surgery with a standard approach
5. Written informed consent to participate

Exclusion criteria are as follow:

- Sized dressings that are not appropriate for the incision/wound
- Known allergy/hypersensitivity to any dressing components
- Polytrauma patients
- Undergoing arthroplasty due to tumor
- Fractures
- Wound at the surgical site prior to surgery
- Neurological deficit of operated side (hemiplegia, *etcetera*)
- Subject has documented skin disease at time of enrolment, as judged by the investigator

### Intervention

**Figure 1:**
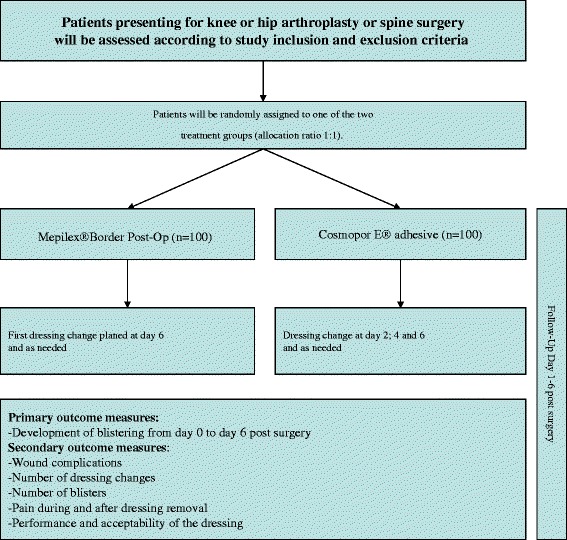
**Flow diagram of the progression through the randomized clinical trial to evaluate the performance of a flexible self-adherent absorbent dressing coated with a soft silicone layer compared to a standard wound dressing after orthopedic surgery.**

The patients will be randomized 1:1 preoperatively as either the Mepilex Border Post-Op group or the standard dressing group (Figure 1). The Mepilex Border Post-Op group will get the first dressing removal 6 days after surgery. The standard wound dressing group will receive dressing removals at day 2, 4 and 6 postsurgery. Both groups will be visited every day postsurgery to allow evaluation and documentation of the appearance of the dressing.

### Mepilex Border Post-Op

Patients in this study group will receive postoperative wound treatment using the Mepilex Border Post-Op dressing (Mölnlycke, Goteborg, Sweden). This is a self-adhesive, absorbent dressing designed for acute wounds. It consists of three main parts. Firstly, there is a flexible and transparent backing film that is highly vapor permeable and waterproof: it is coated with a pressure sensitive water-based acrylic adhesive. Secondly there is a flexible, absorbent wound pad consisting of two layers: an absorbent layer with a perforated pattern for good flexibility, retention, and absorption capacity and a gap layer for high initial absorption and good distribution of exudates. Finally, it also includes a wound contact layer of acrylate adhesive, polyurethane film and soft silicone. The soft silicone layer adheres gently to dry skin surrounding the wound, but not to the moist wound surface. The soft silicone layer is covered with a polyethylene release film. The wound coverage section is white and the adhesive section is transparent to ease placement.

### Standard wound dressing

Patients in this study group will receive postoperative wound treatment using the standard Cosmopor E adhesive, an island wound dressing (Hartmann AG, Heidenheim, Germany).

It is is a sterile, self-adhesive wound dressing made of soft nonwoven polyester, with an absorbent viscose pad covered with a nonadherent porous wound contact layer.

Cosmopor E carries the CE mark according to EU directive 93/42/EEC for medical devices. The product is classified as a class I sterile medical device. A conformity assessment has been performed for Cosmopor E, and it has been shown to be in compliance with all applicable requirements of the above mentioned directive.

### Outcome measures and assessments

The primary outcome measure is blistering incidence from day 0 to day 6 postsurgery. Photo documentation will be evaluated in a blinded manner by the CEC. The secondary objectives will evaluate the dressing performance, comfort and overall acceptability by standardized assessment criteria. The individual pain before, during and after the dressing removal will be documented in a 1 to 10 rating scale equal to the visual analog scale. During the removal of the dressing, we will count the used materials and keep records of the time spent in minutes to summarize the resources and costs in general.

### Sample size

Though Pelet *et al.* [9] reported on a percent blistering reduction from 59% (Mepore Pro) or even 61% (Hypafix) to just 3%, we anticipate a less optimistic scenario, for instance, such as described by Burke *et al.* [10]. They reported a reduction from 17.7% (standard) to 4.8% (Jubilee), which we consider both realistic and clinically relevant. To detect this proportional difference with 80% power and a two-sided type I error of 5%, the uncorrected chi-square test requires 93 patients per group. Accounting for the stratification and any loss to follow-up (we expect none), 100 patients per group will be randomized.

### Randomization

Patients will be randomly assigned to one of the two treatment groups (allocation ratio 1:1). The randomization will be stratified according to surgery type (hip, knee, or spine) and blocked (permuted blocks of varying length). The randomization will be implemented by sequentially numbered, opaque, sealed envelopes containing details of the dressing to be applied.

### Statistical analysis

Primary analysis will be according to the intention-to-treat. A patient is evaluable for this analysis if they underwent surgery and received a study treatment. If the clinical course cannot be fully evaluated, the patient will be considered a treatment failure. Secondary analysis will include all patients essentially treated and observed according to protocol (per protocol set, PPS), that is, no study visit missed, key outcome variables taken.

Primary outcome analysis: The proportion of incident blistering in the two groups will be compared with a two-sided type I error of 5% by the Mantel-Haenszel test, stratified by type of surgery: that is, the null hypothesis H_0_: common odds ratio for blistering = 1 is tested against the alternative hypothesis H_A_: common odds ratio for blistering ≠ 1. A subgroup analysis by type of surgery and gender will be performed. Heterogeneity of treatment effect and 95% confidence intervals (odds ratio, relative risk, and risk difference) will be evaluated by Mantel-Haenszel methods.

Secondary outcome analysis: Pain, number of dressing changes/blisters, and performance/acceptability ratings will be analyzed by rank-based methods, that is, Wilcoxon rank sum/signed rank test. Other wound complications will be assessed by chi-square test (or Fisher’s exact test when required).

## Discussion

Postoperative wound infection is a preventable risk associated with significant adverse outcomes and increased costs of care. Currently, patients are treated with a standard dressing, which is changed every 2 days. A comfortable wound dressing that minimizes the risk of blistering, which will be changed only after 7 days, once the wound edges have closed, could minimize the risks of postoperative superficial wound infections. Because both treatments are acceptable, we can compare them in an attempt to optimize postoperative wound care. Finally, not only the used dressing mentioned in this study can influence the outcome of developing wound blisters and complications. Also the number of dressing changes in general may have a significant influence on the outcome. This would be considered separately in the final examination of the study results.

## Trial status

At the time of manuscript submission, the trial was actively enrolling participants.

## Abbreviations

CEC: clinical evaluation committee
